# Penetrating arterial trauma to the limbs: outcome of a modified protocol

**DOI:** 10.1186/1749-7922-8-51

**Published:** 2013-12-04

**Authors:** Antonio Krüger, Carla Florido, Amelie Braunisch, Eric Walther, Tugba Han Yilmaz, Dietrich Doll

**Affiliations:** 1 Department of Trauma-, Hand- and Reconstructive Surgery, Philipps-University, Baldingerstr. 1, Marburg, Germany; 2 Department of Visceral, Thoracic and Vascular Surgery, Philipps-University of Marburg, Marburg, Germany; 3 Department of Surgery, Izmir, Turkey, Baskent University, Ankara, Turkey; 4 Department of Trauma, Chris Hani Baragwanath Academic Hospital, Johannesburg, Soweto, South Africa; 5 Department of Surgery, St.-Marien-Hospital Vechta, Teaching Hospital of the MHH Hannover University, Vechta, Germany

**Keywords:** Popliteal artery, Penetrating trauma, Treatment protocol, Outcome, Amputation rate, Vascular repair, Vascular surgeon

## Abstract

**Background:**

Penetrating arterial injuries to the limbs are common injuries in high volume trauma centers. Their overall surgical results reported in the literature are satisfactory - apart of those of the popliteal artery that still may lead to a significant incidence in amputations. With the present study we assessed our outcome with penetrating arterial injuries to the limb as to see if the direct involvement of vascular surgeons in the management of popliteal artery injuries leads to an improved (lowered) amputation rate. Results were benchmarked with our published results from previous years.

**Methods:**

All patients sustaining penetrating arterial injuries to the limbs admitted to the Chris Hani Baragwanath Academic Hospital during an 18- month period ending in September 2011 were included in this study. Axillary, brachial and femoral artery injuries were operated on by the trauma surgeons as in the past. All popliteal artery injuries were operated on by the vascular surgeons (new).

**Results:**

There were a total of 113 patients with 116 injuries, as some patients had multiple vascular injuries: 10 axillary, 47 brachial, 34 femoral and 25 popliteal artery injuries. Outcome of axillary, brachial and femoral artery injury repair were excellent and not significantly different from our previous reported experience. Injury to the popliteal artery showed a diminished re-exploration rate from 34% down to 10% (p = 0,049) and a decrease of amputation rate from 16% to 11% which was statistically not significant (p = 0,8).

**Conclusion:**

Penetrating arterial trauma to the axillary, brachial and femoral artery is followed by excellent results when operated by trauma surgeons. In the case of popliteal artery injury operated by the vascular surgeons, the results of this study do not show any statistically significant difference related to amputation rate from our previous reported studies when operated by trauma surgeons. Taking into consideration the diminished re-exploration rate and a tendency to a lower amputation rate, we feel that there is possible tendency of better outcome if operated by vascular surgeons. Multicenter studies with large number of enrolled patients will be required to prove the validity of this suggestion.

## Introduction

Penetrating arterial injuries to the limbs generally show a good outcome if an experienced trauma team operates on them without undue delay. Several articles studying this subject were published from our institution within the last two decades [1-5]. In the last few years we proceeded to certain changes in our management protocol of this type of injury: popliteal artery injuries, formerly done by trauma surgeons, were now done by vascular surgeons. The purpose of this study was to assess the effect of these changes in our management protocols to patient outcome in terms of re-exploration rate as well as the rate of limb loss (amputation).

## Patients and methods

Chris Hani Baragwanath Academic Hospital with approximately 3000 beds is Teaching Hospital of the University of Witwatersrand, as it is the largest hospital in the southern hemisphere. The trauma unit deals with neck, cardiothoracic, abdominal and vascular trauma as well as with polytrauma patients. It is run by general surgeons with a subspecialty in trauma. The hospital services care for approximately 3, 5 million people living in SOWETO (South West Township), Johannesburg, South Africa.

In this study we included all patients with penetrating trauma of the major arteries of the extremities who were admitted to hospital over 18 months (from the 1st of March 2010 to 1st of September 2011. Arterial injuries distal to the bifurcation of the brachial or the trifurcation of the popliteal artery were not included in the study.

Patient variables extracted included gender, age, injury mechanism, admission vital signs, Glasgow Coma Scale (GCS), preoperative investigations, initial management and outcomes. Data were entered into a computerised spreadsheet (Microsoft Excel 2007) and analyzed using SPSS for Windows^©^, version 18.0. Graphic presentation was done by Microsoft Excel 2007 and Graph Pad Prism^©^. Discrete variables are presented as proportions (percentages), unless stated otherwise and were analysed by Fischer’s exact test. Statistical significance was accepted if p < 0, 05.

The majority of the patients sustained gunshot injuries through commercially available handheld low velocity weapons (pistols). Patients with high-velocity weapons contact, as the AK-47 been the most common high velocity weapon used in our society, were rarely seen arriving in the hospitals. Amongst the 61 patients out of the 113 patients who sustained gunshot injuries, it was generally difficult if not impossible to determine the caliber of weapon used and from what distance it was fired.

The trauma surgeon on call is present on the hospital premises at a 24 hour rotation. He is responsible for the management of all patients, from their arrival via the resuscitation room treatment (if needed) to the operating theatre. He is also responsible for the care of patients admitted to ICU or to the trauma ward. All arterial injuries irrespective of the anatomical site are dealt with by the trauma surgeons. The only exception is the popliteal artery injuries which according to our new management protocol are operated by the vascular surgeons.

All patients were admitted and resuscitated in the trauma resuscitation area applying the world wide standardized Advanced Trauma Life Support (ATLS ®) principles. On admission to the trauma resuscitation area all patients – only if haemodynamically stable - received a full body X- Ray examination with a Lodox ® (Low Dose X-Ray) scanner, so that the presence of bullet fragments or fractures could be visualized.

Our protocols stress the importance of emergency room hemorrhage control; direct digital pressure being the most effective method, which was maintained until definitive operative control was established. Balloon tamponade has been a useful adjunctive measure, where one ore more Foley catheters are inserted into the tract of the missile or stab and the balloon inflated with fluid until hemorrhage is controlled. Large skin wounds are rapidly closed around the catheter(s) with skin sutures to prevent dislodgement during balloon inflation and to assist in creating a tamponade.

Physical examination was the cornerstone of the diagnosis and relied mostly on the presence of “hard” or “soft” signs of arterial injury (Tables 1 &2). “Hard” signs are indicative of ischemia or ongoing hemorrhage and include absent distal pulses, extensive external bleeding, expanding or pulsatile hematoma, palpable thrill, continuous murmur, or other signs of distal ischemia (pain, pallor, coolness). The presence of “hard” signs mandated immediate surgical exploration. “Soft” signs of arterial injury included a history of severe bleeding at the trauma scene, nonexpanding hematoma, diminished but palpable pulses, and peripheral neural deficit. Doppler pressure measurements were undertaken in our department as an adjunct to stratify risk in patients with arterial trauma. In the absence of “hard” signs, a Doppler pressure deficit of greater than 10 per cent, compared with the contralateral limb, was considered a “soft” sign of arterial injury. As recommended by Frykberg et al. and confirmed in our previous published experience, proximity of injury to major vessels was not considered a “soft” sign [6].

**Table 1:** Hard clinical signs in n = 113 patients with arterial vascular injuries

Clinical signs*	**Femoral**		**Popliteal**		**Axillary**		**Brachial**		**Total**	
	**all pts: n = 34**		**all pts: n = 25**		**all pts: n = 10**		**all pts: n = 47**		**all pts: n = 113**	
	**pts [n]**	**pts [%]**	**pts [n]**	**pts [%]**	**pts [n]**	**pts [%]**	**pts [n]**	**pts [%]**	**pts [n]**	**pts [%]**
Cold ischemic extr.	8	24%	18	72%	2	20%	11	23%	39	35%
Absent pulses	14	41%	14	56%	7	70%	19	40%	54	48%
Bruit or thrill	1	3%	0	0%	0	0%	0	0%	1	1%
Exp. or pulsating H	3	9%	2	8%	0	0%	2	4%	7	6%
Pulsatile bleeding	6	18%	5	20%	3	30%	12	26%	26	23%

Seven of the patients underwent immediate amputation. *Please note that multiple signs are possible. Pts = patients; extr. = extremity; Exp. or pulsating H. = patients with expanding or pulsating hematoma.

**Table 2:** Soft clinical signs in n = 113 patients with arterial vascular injuries

Clinical signs*	**Femoral**		**Popliteal**		**Axillary**		**Brachial**		**Total**	
	**all pts: n = 34**		**all pts: n = 25**		**all pts: n = 10**		**all pts: n = 47**		**all pts: n = 113**	
	**pts [n]**	**pts [%]**	**pts [n]**	**pts [%]**	**pts [n]**	**pts [%]**	**pts [n]**	**pts [%]**	**pts [n]**	**pts [%]**
Nonexpanding H.	7	21%	1	4%	2	2%	3	6%	13	12%
Paraesth./Paresis	6	18%	6	24%	6	60%	17	36%	35	31%
Decreased pulses	5	15%	3	12%	1	10%	11	23%	20	18%

Seven of the patients underwent immediate amputation. *Please note that multiple signs are possible. Pts = patients; Nonexpanding H. = patients with nonexpanding hematoma; Paraesth./Paresis = paraesthesia and / or paresis of the extremity in the awake patient.

**Figure 1:**
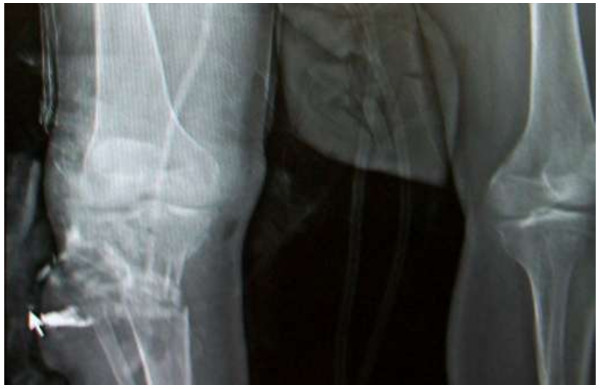
**Transection of the right popliteal artery at the level of the trifurcation after gunshot injury (Lodox picture).** Bullet fragment can be seen right to white arrow.

According to our previous recommendations the most reliable tool for detection of arterial injury was the arteriography. This slowly changed over the years with the use of multi-slice CT scanners. According to our new protocol we are performing only CT- arteriography if this is indicated by the clinical presentation. Patients with “soft” signs of vascular injury underwent CT- arteriography with a 64 or 128 detector row CT scanner if hemodynamically stable. CT- arteriography was also performed on physiologically stable patients if there was uncertainty regarding the site of injury, e.g., multiple gunshot wounds or shotgun wounds. If the patient requiring arteriography was physiologically too unstable to be transferred to the CT scanner (approximately 50 meters from our trauma resuscitation area), then arteriography was carried out in the trauma resuscitation area with the use of the Lodox - Scanner (Figure 1) or preoperatively in theatre with a C- Arm.

All patients were given a dose of Cefazolin 1 g. intravenously perioperatively, and the dose was administered every 12 hours for a total of 48 hours. In patients with associated abdominal injury the antibiotic regime consisted of Amoxicillin-Clavulanic acid 1,2 g. intravenously.

After operative exploration of the injured artery we proceeded with the debridement of the site of the injury. The distal part of the vessel routinely underwent thrombectomy with a Fogarty catheter to ensure sufficient backflow. Primary repair or primary anastomosis was practiced if it was not leading to any narrowing to the injury site or to undue anastomotic tension. If narrowing or tension were pending, a graft was inserted. Although an autologous saphenous vein graft from the contralateral site was our first choice, PTFE (Polytetrafluoroethylene) graft was used if the saphenous vein was unavailable, of the vein was of insufficient diameter or if the time needed to harvest the vein would be detrimental to the patient’s outcome. Whenever graft was used, great care was taken to cover it with viable muscle or other well perfused soft tissues available. In most cases venous injuries were dealt with by ligation. In cases of injury of large diameter veins which could be repaired by simple suturing, ligation could be avoided. We never attempted to repair any venous injuries by complex techniques, such as fashioning of a spiral graft. In all cases venous repair preceded the arterial one.

**Figure 2:**
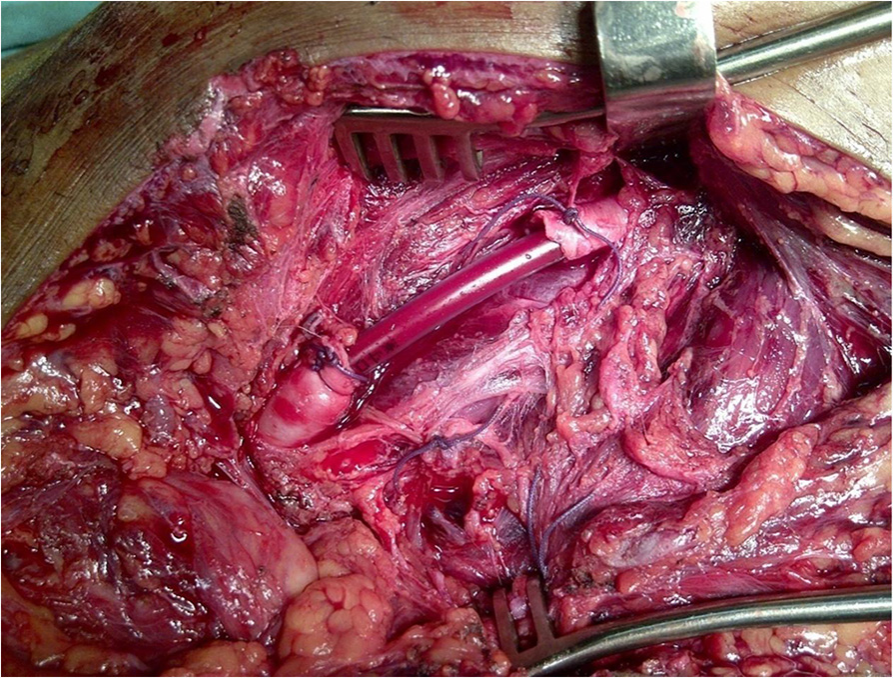
Temporary shunting of the femoral artery.

In cases of skeletal injury accompanied by significant bone instability or length shortening, distal revascularisation was initially achieved by the use of a temporary arterial shunt. In these cases, skeletal fixation followed immediately, as did removal of the temporary shunt and replacement of it by a vein or PTFE graft. Temporary shunting (Figure 2) also was used in cases of physiologically instability of the patient which enforced postponement of definitive management of the injured (damage control situations; pending or obvious DIC).

Early fasciotomy was performed in the presence of distal swelling, severe distal muscular- skeletal injury, delayed restoration of blood flow (more than 4 to 6 hours after accident/injury) and venous ligation. There was a tendency to perform fasciotomy in any doubtful cases or in the presence of an anticipated reperfusion injury [7]. Compartment syndrome was clinically diagnosed and at no stage intra-compartmental pressures were measured.

Nerve injury was repaired at the time of the arterial repair only if the patient was haemodynamically stable and the repair of the nerve was considered technically easy [8].

Methodologywise, in three patients with bilateral femoral arterial injury (with side different treatment and outcome), each side was treated, analyzed and counted as a single injury.

## Results

There were a total of 113 patients who underwent operation for 116 penetrating arterial injury to the limbs. There were 103 male and 10 female patients. The mean age was 25 years (range 13–66 years). Of these 113 patients, 61 had received gunshot wounds and 30 received stab knife wounds. 20 injuries were inflicted by other sharp instruments and in two patients injury was related to dog bites. There were 10 axillary artery, 47 brachial artery, 34 femoral artery (15 common, 14 superficial and 5 profunda femoral artery) and 25 popliteal artery injuries. Three patients had simultaneous profunda femoral and superficial femoral artery injury.

Fifty-nine out of the 113 (52%) patients who underwent operation presented with additional trauma to other anatomical areas including bones / fracture dislocations and nerve lesions.

Tables 3 and 4 illustrate the operative findings and the type of arterial repair done depending on the site of the injury.

**Table 3:** Intraoperative findings in n = 113 patients with arterial vascular injuries

Intrap. findings*	**Femoral**		**Popliteal**		**Axillary**		**Brachail**		**Total**	
	**all pts: n = 34**		**all pts: n = 25**		**all pts: n = 10**		**all pts: n = 47**		**all pts: n = 113**	
**Artery**	**pts [n]**	**pts [%]**	**pts [n]**	**pts [%]**	**pts [n]**	**pts [%]**	**pts [n]**	**pts [%]**	**pts [n]**	**pts [%]**
Thrombosed	3	9%	1	4%	3	30%	3	6%	10	9%
Fully transsected	17	50%	19	76%	5	50%	28	60%	69	61%
incompletely transs.	11	32%	5	20%	4	40%	11	23%	31	27%
Dissected	2	6%	0	0%	0	0%	4	9%	6	5%
AV fistula	2	6%	0	0%	0	0%	1	2%	26	23%

*Please note that multiple signs are possible. Pts = patients; AV fistula = traumatic arterio-venous fistula discovered.

**Table 4:** Intraoperative vascular procedures done in n = 113 patients with arterial vascular injuries

**Vasc. Procedure**	**Femoral**		**Popliteal**		**Axillary**		**Brachial**		**Total**	
	**all pts: n = 34**		**all pts: n = 25**		**all pts: n = 10**		**all pts: n = 47**		**all pts: n = 113**	
	**pts [n]**	**pts [%]**	**pts [n]**	**pts [%]**	**pts [n]**	**pts [%]**	**pts [n]**	**pts [%]**	**pts [n]**	**pts [%]**
Lateral arteriorraphy	2	6%	0	0%	0	0%	1	2%	3	3%
Primary end-to-end	3	9%	2	8%	2	20%	15	32%	22	19%
Vein interpositiona	12	35%	17	68%	5	50%	28	60%	62	55%
PTFE interposition	12	35%	0	0%	2	20	0	0%	114	12%
Shunt/Stent	1	3%	1	4%	1	10%	2	4%	5	4%

Pts = patients; PTFE = Poly-tetra-fluoro-ethylen interposition graft.

Sixteen of these 59 patients (27%) with additional injuries were hypotensive with a systolic BP < 90 mm Hg on admission. In contrast to them, only 10 patients of the 54 patients without concomitant injuries (19%) presented with systolic hypotension.

### Limb-saving surgery

113 patients were receiving an operation, and 92 (81%) of them had a successful primary reconstruction. This were all patients with axillary artery injury, 40 out of 46 (87%) patients with brachial artery injury, 24 out of 30 (80%) patients with femoral and 18 out of 20 (90%) patients with popliteal artery injury. There were 12 (11%) patients who developed complications related to the initial interposition graft (bleeding, thrombosis); all of them were re-explored. All re-explorations were performed by the trauma surgeon in charge.

### Brachial artery results

Of the 47 patients with brachial artery injury, one already presented with severe ischemia of the forearm and he underwent primary amputation for already overt muscle necrosis. Of the 46 patients who underwent brachial artery repair or graft, 6 (13%) patients had to be re-explored. These were the 2 patients with saphenous vein interposition graft who developed thrombosis and 2 patients with basilic vein interposition graft who developed postoperative bleeding. The use of basilic vein graft was a diversion to our protocol. A new saphenous vein graft was used in all four cases with satisfactory result. Another patient with saphenous interposition graft had to be taken back to theatre for postoperative bleeding from the anastomosis site that was controlled with stitches. Another patient developed thrombosis in a feeding tube which was used as a temporary emergency shunt. All 46 patients operated with brachial artery injury were discharged with a good radial pulse (Table 5).

**Table 5:** Results and outcome of surgical therapy

		**Femoral**		**Popliteal**		**Axillary**		**Branchial**		**Total**	
		**all inj: n = 34**		**all pts: n = 25**		**all pts: n = 10**		**all pts: n = 47**		**all pts: n = 113**	
**Outcome**		**pts [n]**	**pts [%]**	**pts [n]**	**pts [%]**	**pts [n]**	**pts [%]**	**pts [n]**	**pts [%]**	**pts [n]**	**pts [%]**
Immediate amputation		1	3%	4	16%	0	0%	1	2%	6	5%
DCS amputation		0	0%	1	4%	0	0%	0	0%	1	1%
Revisions	total	6	18%	2	8%	0	0%	6	13%	14	12%
	successful	1	3%	0	0%	0	0%	6	13%	7	6%
	amputation	5	15%	2	8%	0	0%	0	0%	7	6%
Long ischemia & amputatio		3	9%	12%	0	0	0%	0	0%	3	3%
Deaths		3	9%	0	0%	1	10%	1	2%	5	4%
Successful repair		29	85%	18	72%	10	100%	46	98%	103	91%

DCS amputation = vascular repair was aborted because of trauma load leading to damage control procedures. All deaths were due to trauma severity and consecutive DIC. Death does not exclude a good vascular result, while amputation does. Patent vascular repair with good flow before death - without pending amputation - were judged a good result. Pts = patients.

### Femoral artery results

One grossly avital limb which was amputated straight away was not calculated as treated or treatment failure (early amputation). There were overall 6 out of 34 (18%) cases with femoral artery injury that had to be re-explored, 3 of them were associated with initially delayed presentation (approximately 12 hours post injury) and with pulseless cold limb. They were all referred from one smaller district hospital to our hospital. These three had all unsuccessful re-exploration that led to amputation. One of these patients died after repeated amputations. Of the other three patients one had successful re-exploration and two others underwent amputation. Therefore 5 of 33 femoral artery injuries underwent amputation after unsuccessful primary reconstruction, an overall amputation rate of 15%. If we exclude the 3 patients who were transferred to us from the other hospital with an approximately 12 hours post injury delay and signs of severe ischemia, there were only 2 amputations out of 30 cases of adequately treated limb injuries of the femoral arterial axis (7%; Table 5).

### Popliteal artery results

4 of the 25 patients with popliteal artery injury (16%) underwent immediate amputation as muscles were found to be not viable during 4-compartment-fasciotomy. A fifth patient also underwent amputation as he was physiologically unstable and it was not possible to use an arterial shunt, with the injury being very close to the trifurcation, so the popliteal artery needed to be ligated proximally. This patient developed severe ischemia of the leg that was amputated at a second stage. Two patients with saphenous vein grafts developed complications regarding thrombosis or insufficient reperfusion of the limb. They were explored unsuccessfully and finally underwent limb amputation. Therefore, of the 20 patients with popliteal artery injury that underwent arterial grafting, 2 underwent amputation, with an amputation rate of 10% (Table 5).

### Additional injuries

Seven patients had an exploratory laparotomy because of concomitant abdominal injury. Abdominal surgery preceded the vascular repair in 4 times, whereas limb surgery was done in 3 patients. Abdominal surgery preceded limb surgery in cases of life threatening abdominal hemorrhage. There was concomitant bone injury in 32 out of 113 (28%) patients, two out of 10 (20%) in the axillary group, eight out of 47 (17%) were in the brachial group, six out of 34 (18%) were in the femoral group and 16 out of 25 (64%) in the popliteal group. Fourteen of those patients required external fixation, 1 in the axillary, 3 in the brachial, 3 in the femoral and 7 in the popliteal group.

There were 33 out of 113 (29%) patients documented with additional nerve injury - one out of 10 (10%) with axillary, 29 out of 47 (62%) with brachial and three out of 25 (12%) with popliteal artery injury.

There was a 31% overall venous trauma rate with 35 concomitant vein injuries.

Compartment syndrome was clinically diagnosed and at no stage intra-compartmental pressures were measured. As fascial compartment measures are known to be notoriously unreliable, fasciotomy was done on the base of clinical judgment alone. Four out of 47 (9%) patients with brachial artery injury, 9 out of 31(29%) patients with femoral artery injuries and 6 out of 25 (24%) patients with popliteal artery injuries already presented compartment syndrome at the time of admission. Early full- thickness fasciotomies were performed in 2 out of 10 (20%) patients with axillary, 20 out of 47 (43%) with brachial, 8 out of 31 (26%) patients with femoral and 17 out of 25 (68%) with popliteal artery injuries.

There was an average of 22% incidence of postoperative wound infection, with no significant late morbidity. This was unrelated to the anatomical site of the injury.

### Mortality

There were five postoperative deaths, of whom were 2 deaths following femoral artery injury. Another patient with gunshot injuries to the abdomen and femoral artery underwent damage control laparotomy and shunting of the artery (Figure 2). He had to be re-taken to theatre 16 hours later for relook laparotomy. There was no specific bleeding source found, which was due to DIC. The arterial shunt was left in place and the patient demised the next day in ICU from disseminated intravascular coagulopathy. One patients with injury to the brachial artery went into cardiac arrest intraoperatively. He was successfully resuscitated and operated; unfortunately he demised postoperatively. There was one patient with stab wounds to the axillary artery, neck, chest, abdomen and lower extremities who developed DIC and demised postoperatively in ICU at the day of admission.

Thus concomitant trauma to neighbouring organ regions outweighed the vascular trauma in terms of mortality by far.

## Discussion and conclusion

Over the last 20 years there has been a gradual reduction in the incidence of penetrating trauma presenting in our hospital, with a corresponding reduction of penetrating arterial injuries. In 1994 the incidence of penetrating trauma presenting at the Chris Hani Baragwanath Academic Hospital was 95% compared to 5% of blunt trauma. In 2008 the incidence of penetrating trauma was 47% compared to 53% of blunt trauma. As penetrating trauma is directly related to crime, it would seem that crime in Soweto has diminished over the years.

The reason for this is three fold: Firstly, the establishment of democracy led to the disappearance of political violence. Secondly, there are more employment opportunities for the previously disadvantaged population groups. Thirdly, the population now considers police as their protector and not as an oppressior of the Apartheid regime, this leading to increased population - police cooperation.

Another change that has developed over the years is that there are more patients referred from the district hospitals that are covered by our hospital. This results in a significant number of patients with delayed presentation, leading to a considerable number of primary amputation or thrombotic postoperative complications in this group of patients.

Diagnosticwise, the use of CT arteriography (CTA) has completely replaced the conventional “invasive” arteriography in our hospital and has greatly facilitated the investigations of arterial trauma. In our experience it has been satisfactory in all cases and it there was never any need to perform conventional arteriography. Hitherto, especially if there is clinical presence of hard symptoms of vascular injury, the positive predictive value is close to 100% [9]. Mindbogglingly, infrapopliteal vasospasms have not been found in surgical explorations with pathological CTA.

The mortality within our patient group is 5/113 patients, with 3 deaths attributed to DIC and coagulopathy. It may be pointed out that associated penetrating trauma to nerves, veins, and other body regions are still not uncommon in South Africa. We noticed a relatively small incidence on nerve injury in popliteal injuries in our collective (12%), which is said to ultimately to determine the functional outcome of the limb [10,11]. If we compare our patients’ trauma with penetrating injuries from other studies, 2/3 of all penetrating vascular injuries here are gunshot-related, where others studies are dominated by stab injuries [12,13]. Thus the force and destruction to the neurovascular bundle, bones, soft tissues and remote body regions should have been expected to be substantially higher in our study [14]. Indeed, 32 of our 113 patients arrived with combined vascular and bony injuries, among them the highest incidence at 60% of all patients in the popliteal group. Thus the high amputation rate in the popliteal group of 7/25 (4 primary amputations, one amputation related to hemodynamic instability of the patient and 2 late amputations) is not surprising.

The mean time between injury and operation in our previous reported experience as well as in our present are comparable. It was thus interesting to compare our previous experience outcome on each different anatomical site of injury with the actual results and with the literature. As pointed out, isolated vascular injury may come with an amputation rate as low as 3% [15], but penetrating trauma, increased transport times (longer warm ischemia time) and coagulopathy may push the amputation rate up to 33% and higher [16], as do combined arterio-venous trauma, fractures [17,18], hypotension and torso injuries increase mortality [19].

Comparing brachial, popliteal and femoral mortality, the latter will be the highest (3/34), as the proximal femoral vessel has the highest flow, no collaterals, may not easy be assessable for bleeding with tourniquet and may come as multiple vascular injury, as was present in three of our femoral patients.

Focussing on the arterial injury of the upper limb, we see that the overall outcome in the past and the present studies is very satisfactory particularly in the present study: all operated patients with axillary and brachial injuries had successful outcome. The same applies for the patients with femoral artery injury if we do not take into consideration the 3 patients who were referred from other hospital to us with a more than 12 hours delay between injury and surgery. In all the studies (previous and present) reported from our institute, the injuries were operated by trauma surgeons.

In contrast to that, if we compare our patients outcome for gunshot popliteal artery injury, we see that there is a difference between our present and our past reported experience. Previously the amputation rate of the combined experience of this type of injury was 11 out of 68 (16%), not considering the primary amputations [20]. At our present study again taking into consideration only the gunshot injuries to the popliteal artery (21 out of 25 patients of our study), there were 2 out of 18 patients (11%) who underwent amputation. Again we did not include patients with primary amputation due to muscle necrosis on arrival in this calculation. All the penetrating popliteal artery injuries not caused by gunshot wound had a positive outcome. So the amputation rate of the present study compared with the old ones is 11% to 16% (p-value = 0, 8). It is also interesting to see that the number of re explorations in the past experience is 23 out of 68 patients (34%) compared to the present experience that is 2 out of 18 patients (11%), which just touches the statistical level of significance with p = 0,049.

Knowing that the overall injury to operation time interval between the 2 groups has been comparable, we have the impression that our present results are better than those of the past. The patients in the older study were operated by the trauma surgeons. In the recent study - because of the change of management protocol - the injury in this specific popliteal site was operated by the vascular surgeons. This is the only parameter that would logically lead to a difference in outcome.

Patients presenting with penetrating arterial injuries are in their great majority young men and, to a lesser extent, woman. As a consequence their arteries are of good quality. Particularly with arteries of the upper limb and the femoral artery, there is a significant network of collaterals that overall contribute to satisfactory outcome, by providing critical distal blood supply and many times keeping muscle viability for a considerable length of time. These factors can lead us to the conclusion that the operations in young people at these sites are not only technically easier due to the good quality of the arteries but are also probably forgiving minor technical imperfections. This is not the case with the popliteal artery, particularly the distal one that is not supported by an extensive collateral network. A further “aggravating” factor at this site is the difficulty in access and position of the graft. Taking into consideration the above characteristics of the popliteal artery and our significantly improved results after the change of our protocol management, we are tempted to assume that this change is due to the fact that patients were operated by vascular surgeons. At the end of the day they are more experienced in dealing with difficult vascular operative situations.

Four patients with popliteal artery injuries in the authors’ recent experience underwent immediate amputation. Perhaps this fact alone accounted for the small improvement in outcomes. By increasing the rate of early amputations, this might reduce the number of graft failures and late amputations as the result of a more favourable selection bias. This fact could also have accounted for the better results rather than “better technique” employed by the vascular surgeons.

The remaining question arising from our results is: should all patients with arterial trauma to the limbs be operated by vascular surgeons? Our opinion is that they should not, taking into consideration our results with the axillary, brachial and femoral artery injuries. This is supported by the international literature as well that reports excellent results with this type of injury.

We are therefore convinced that patients with penetrating trauma to the axillary, brachial and femoral arteries are getting excellent service when operated by trauma surgeons of a Level I Trauma centre. On the other hand we feel that popliteal arteries, particularly the distal ones, should be operated by vascular surgeons through the trauma service.

In conclusion penetrating trauma to the arteries of the limbs is an injury that should be dealt with as an absolute emergency. In the presence of “soft” signs of arterial injury, the use of new generation spiral CT- scanners leads to excellent diagnostic results, compared to those of arteriography. The outcome with axillary, brachial and femoral artery injuries - when operated by experienced trauma surgeons - are satisfactory. When it comes to popliteal artery injury there is a statistically significant reduced rate of popliteal artery re-exploration if vascular surgeons do the primary repair. Thus we believe it is related to better surgical technique, due to the involvement of the vascular surgeons. There is a higher percentage – although not statistically significant rate - of limb salvage with vascular surgeons and popliteal repair. We are wondering if a study with a larger number of patients will lead to a statistically significant reduction of amputation rate. We therefore feel that this issue should further be explored through a multi-center study so that we come to a solid and universally acceptable conclusion, related to our suggestion that popliteal artery injury should rather be operated by vascular and not trauma surgeons.

## Competing interests

The authors declared that they have no competing interests.

## Authors’ contributions

Conception and design: DD, CF. Acquisition of data: CF, AB, EW. Statistical analysis: CF. Analysis and interpretation of data: CF, DD, AK. Drafting the article: DD, CF, AK. Critically revising the article: all authors. All authors read and approved the final manuscript.
